# Effects of the *Agaricus bisporus* stem residue on performance, nutrients digestibility and antioxidant activity of laying hens and its effects on egg storage

**DOI:** 10.5713/ajas.19.0853

**Published:** 2020-02-25

**Authors:** Bowen Yang, Guoxian Zhao, Lin Wang, Shujing Liu, Jie Tang

**Affiliations:** 1 College of Animal Science and Technology, Hebei Agricultural University, Baoding 071000, China

**Keywords:** *Agaricus bisporus*, Antioxidant Activity, Digestibility, Egg Quality, Laying Hens, Laying Performance

## Abstract

**Objective:**

The purpose of this experiment was to investigate the effects of the *Agaricus bisporus* stem residue (ABSR) on the performance, nutrients digestibility, antioxidant activity of laying hens, and its effects on egg storage to determine the appropriate dosage of ABSR, so as to provide a scientific basis for the effective utilization of ABSR.

**Methods:**

A total of 384 53-wk-old Nongda III layers were randomly divided into six treatments, four replicates in each treatment and 16 birds in each replicate. The control treatment was fed with basic diet, while experimental treatments were fed with diets of 2%, 4%, 6%, 8%, and 10% ABSR respectively. The experimental period was 56 d.

**Results:**

The results showed that compared with the control treatment, ABSR had no significant effect on laying performance (p>0.05). The crude protein and total energy digestibility of experimental treatments was significantly higher than those of control treatment (p< 0.05). When eggs were stored for 1 wk, 2 wk, and 3 wk at 25°C, there were no significant differences in egg storage between the experimental treatments and the control treatment (p>0.05). The superoxide dismutase (SOD) activity and glutathione peroxidase (GSH-Px) activity in the serum of the experimental treatments were significantly higher than those of the control treatment (p<0.05), and the malonaldehyde (MDA) content did not change dramatically. SOD activity in yolk of experimental treatments was significantly higher than that in control treatment (p<0.05); MDA content in yolk was markedly lower than that in control treatment (p<0.05). The activity of GSH-Px and SOD in yolk of experimental treatments was significantly higher than that of control treatment stored at 25°C for 21 d, and the content of MDA was significantly lower than that of control treatment (p<0.05).

**Conclusion:**

ABSR can be used to improve the antioxidant activity of laying hens without affecting laying performance.

## INTRODUCTION

*Agaricus bisporus* (*A. bisporus*), belonging to basidiomycetes, was one of the commonly cultivated mushrooms, and has the characteristics of low cost, delicious taste and rich nutrition. The stem residue of *A. bisporus* accounted for 10% to 13% of the total mushroom weight, and it has been proved that the *A. bisporus* stem has a similar nutrients composition as its cap [1]. *A. bisporus* stem residue (ABSR) is usually treated as waste during processing, which might result in waste of resource and environmental pollution.

Reactive oxygen species (ROS) are produced by the normal metabolism of aerobic cells and are the strongest oxidants in the body [2]. A small amount of ROS can participate in signal transduction and transcription regulation as signal molecules [3], while excessive ROS can cause lipid peroxidation, damage DNA or induce apoptosis. Heavily producing laying hens can almost produce an egg every day, so their metabolism of energy and lipid is vigorous. With the increase of age, the ability of scavenging free radicals decreases, which leads to the accumulation of ROS. ROS accumulation will cause metabolic oxidative damage, lipid peroxidation and oxidative stress. The most direct consequence is the decline of production performance, and even the occurrence of diseases. Oxidative stress is one of the fundamental factors that affect the performance and health of laying hens. Therefore, many studies focused on how to improve the antioxidant capacity of laying hens.

As people pay more attention to food safety and health, the pursuit of natural additives becomes more and more intense. Consequently, more studies have focused on improving the antioxidant capacity and immunity of laying hens by feeding some natural functional ingredients or plants.

The edible fungi had been well researched due to their effect on disease prevention and treatment, such as antioxidant, immune enhancement, anti-inflammatory, and hypolipidemic [4]. Besides the health benefits, edible fungi also showed outstanding effects on poultry production. Some published studies reported that mushrooms have a positive impact in birds as they can improve production performance, ameliorate microbial population structure in the gut, modulate immune response, enhance antioxidant activity, influence intestinal morphology and improve lipid profile [5–7].

The effective utilization of ABSR in production is still limited. Therefore, in order to make full use of the *A. bisporus* stem residue, it was used as an unconventional feed material replacing part of corn and soybean meal in formulating a feed formula for laying hens. The effects of ABSR on laying performance, digestibility of nutrients, antioxidant activity of laying hens’ serum and yolk and its effects on egg storage were investigated.

## MATERIALS AND METHODS

The protocol was approved by the Animal Care and Use Committee of Hebei Agricultural University. All efforts were devoted to reducing birds’ suffering.

### Experimental products

Mushroom (*A. bisporus*) stem residue was provided by mushroom growers (Chengde, China). Mushroom stem residue was processed by air-drying at about 65°C for approximately 8 hours and ground through a 425 μm sieve prior to being used as a supplement to basal rations in the experimental trials. The nutrients in ABSR are shown in Table 1.

### Experimental animals and management

A total of 384 53-wk-old Nongda III layer (white dwarf layer) were purchased from BAU Breeding Poultry Co., Ltd. (Beijing, China). Birds were raised in closed, mechanically ventilated barn containing stainless steel cages, two birds per cage. The cage size was approximately 45 cm×45 cm×45 cm. Birds were allowed 16 h of light (10 to 20 lux) a day during the experiment. Feed and water were provided *ad libitum*. The room temperature was kept at about 25°C±2°C. The relative humidity in the barn was kept between 55% to 65%. Disinfection and vaccination were carried out according to Nongda III layers’ feeding management manual.

### Experimental design and diets

The layers were acclimated on a basal diet for 7 d and then randomly divided into 6 treatments with four replicates in each group and 16 birds in each replicate. The control treatment (I) was fed with basal diet, while experimental treatments (II–VI) were fed with diets of 2.0%, 4.0%, 6.0%, 8.0%, and 10.0% ABSR application level respectively. The experimental period was 56 d (from 53-wk-old to 62-wk-old). All treatment diets contained all nutrition required for laying hens according to the National Research Council [8] and feeding standard of chickens in China [9]. The dietary composition and nutrient level are shown in Table 2.

### Laying performance

In the experiment, the number and weight of eggs were recorded accurately daily, and the hen-day egg production and average weight of eggs were calculated. The feed intake was recorded weekly, and daily intake and feed conversion ratio (FCR) were calculated. The FCR was calculated as feed intake divided by egg mass respectively.

### Apparent digestibility

Fecal samples were collected every 24 h during the last three consecutive day before the end of the experiment (collected approximately 100 g/pen one day, 300 g/pen in three days in all). After collection, 10% hydrochloric acid was used for nitrogen fixation. Samples were dried at 65°C for 48 h, then exposed to air at room temperature for 24 h to prepare air-dried samples. Fecal samples were ground through a 425 μm sieve for analysis. Apparent digestibility was determined by the indicator method using acid insoluble ash (AIA) as indicator. Crude protein (CP) was determined by Kjeldahl nitrogen analyzer (Kjeltec 8400, FOSS Analytical Co. LTD, Hillerød, Denmark). The total energy was determined with an oxygen bomb calorimeter (YX-ZR TypeA, Changsha Youxin Instrument Manufacturing Co., Ltd, Changsha, China). The content of calcium (Ca) in feed and fecal samples was determined by potassium permanganate method (GB/T 6436-2018, China). The content of total phosphorus (P) feed and fecal samples was determined by spectrophotometry (GB/T 6437-2018, China). The apparent digestibility is calculated according to the following formula. Apparent digestibility (%) = 100×(1−[A/B]×[C/D]). In the formula, A is the nutrient content in feces; B is the nutrient content in diet; C is the AIA content in diet; D is the AIA content in feces.

### Egg quality characteristics

At the end of the experiment, 12 eggs were randomly selected from each replicate to determine the egg quality. Eggs were stored at 25°C for 1 wk, 2 wk, and 3 wk. Three eggs from each replicate were selected every week to determine its chamber height, albumen height, Haugh units, thick albumen content. Haugh units and albumen height were measured by the Egg Analyzer (Orka Food Technology Co., Ltd, Ramat HaSharon, Israel). The yolk and albumen were separated, and the weight of albumen (accurate to 0.01 g) was weighed by electronic balance; then the whole albumen was sifted through 425 μm sieve for five minutes, and the filtered albumen was called thin albumen, then weighed (accurate to 0.01 g). Thick albumen weight (g) = albumen weight - thin albumen weight. Thick albumen ratio (%) = thick albumen weight/albumen weight×100%.

### Antioxidant indices

At the end of the experiment, two chickens were randomly selected from each repetition. After 12 h fasting with free water supply, the blood was collected by venipuncture under the wing into a 10 mL anticoagulant-free Vacutainer tube (Greiner Bio-One GmbH, Kremsmunster, Austria). After centrifugation at 3,000 rpm for 15 min to obtain serum. Serum samples were put into −20°C for analysis.

The content of malonaldehyde (MDA), superoxide dismutase (SOD) activity and glutathione peroxidase (GSH-Px) activity in serum were determined. Eggs were stored at 25°C for 0 d and 21 d. One egg yolk was taken from each replicate to determine content of MDA, SOD activity and GSH-Px activity.

The activity of GSH-Px and SOD were determined by reduced glutathione-dithiodinitrobenzoic acid method and xanthine oxidase method. The content of MDA was determined by thiobarbituril method. The antioxidant index was determined by assay kit (Nanjing Jiancheng Bioengineering Institute, Nanjing, China). The operation was carried out according to the instructions of the kit.

### Statistical analysis

SPSS 19.0 program (SPSS INC., Chicago, IL, USA) was used to process the analysis. One-way analyses of variance was used to analyze variance. The pen was the experimental unit. Multiple comparisons among different treatment groups were tested by Duncan multiple range test. The results were shown as means. The level of statistical significance was set at p<0.05. Linear and quadratic significance were evaluated by regression estimation in regression procedure. When the linear or quadratic p<0.05, the regression equation is generated. According to the curve, the most suitable ABSR supplement level was calculated. In the quadratic regression equation (Y = AX^2^+BX+C), when Y reaches the maximum value, X = −B/2A.

## RESULTS

### Laying performance

The laying performance results are shown in Table 3. Compared with the control treatment, the hen-day egg production, average egg weight and feed conversion ratios had no significant change (p>0.05). There were obvious differences in daily feed intake between hens fed diets containing 6.0% ABSR and 10.0% ABSR (p<0.05).

### Apparent digestibility

From Table 4 we can see the result of the apparent nutrients digestibility. Supplementing ABSR did not seem to affect the digestibility of Ca and P (p>0.05). But when it comes to CP digestibility, hens treated with 2.0% ABSR diet were prominently higher than those in the control treatment (p<0.05). Meanwhile, the total energy digestibility of hens fed diets containing 2.0% ABSR, 4.0% ABSR, 6.0% ABSR, and 8.0% ABSR was significantly higher than those of the control treatment (p<0.05).

According to the quadratic p-value of apparent total energy digestibility (p<0.05), the relationship between total energy digestibility and the supplement level of ABSR was obviously in a quadratic regression relationship. Therefore, the apparent digestibility of the total energy was used as the independent variable for the quadratic curve fitting, and the results are shown in Table 5. According to the quadratic regression equation, when 5.261% of ABSR was added, the apparent digestibility of total energy reached the highest value.

### Changes of egg quality with storage time

**Figure 1:**
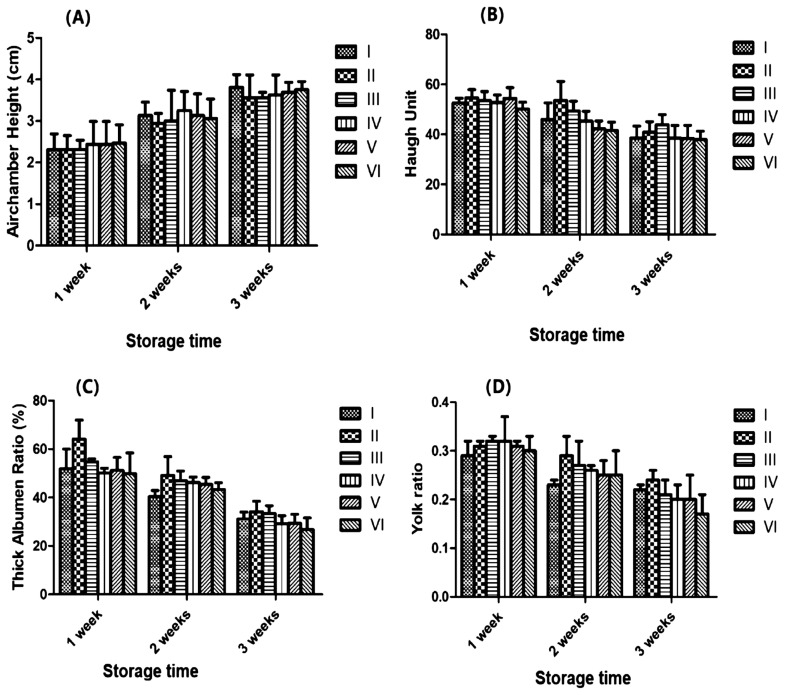
I-0 *Agaricus bisporus* stem residue (ABSR) supplement. II-2.0% ABSR supplement. III-4.0% ABSR supplement. IV-6.0% ABSR supplement. V-8.0% ABSR supplement. VI-10.0% ABSR supplement. (A) Airchamber height in three periods (1 wk, 2 wk, and 3wk) of treatments (mean and standard deviation [SD], n = 12). (B) Huagh unit in three periods (1 wk, 2 wk, and 3wk) of treatments (mean and SD, n = 12). (C) Thick albumen ratio in three periods (1 wk, 2 wk, and 3wk) of treatments (mean and SD, n = 12). (D) Yolk ratio in three periods (1 wk, 2 wk, and 3wk) of treatments (mean and SD, n = 12).

Figure 1 shows the effects of dietary ABSR on the air chamber height, albumen height, Haugh unit, thick albumen ratio and yolk ratio of eggs stored for 1 wk, 2 wk, and 3 wk. Although with the passage of time, each index had increased or decreased, but the change between each experimental treatment and the control treatment was not notable (p>0.05).

### Antioxidant indices

From Table 6, the effect of ABSR on serum GSH-Px activity of laying hens can be seen. Treatment with 4.0% ABSR and 6.0% ABSR diet were markedly higher than that of the control treatment (p<0.05). Because of the quadratic p-value of GSH-Px (p<0.05), the activity of GSH-Px in serum was used as independent variable to fit the quadratic curve. The results were shown in Table 5. When the amount of ABSR was 4.971%, the GSH-Px activity in serum reached the maximum.

Among the effects of ABSR on SOD activity in serum of laying hens, hens fed with 2.0%, 4.0%, 6.0%, and 8.0% ABSR diet were significantly higher than that of the control treatment (p<0.05); treatment with 10.0% ABSR diet did not dramatically differ from the control treatment. The ABSR had no significant effect on serum MDA content in laying hens (p>0.05).

**Figure 2:**
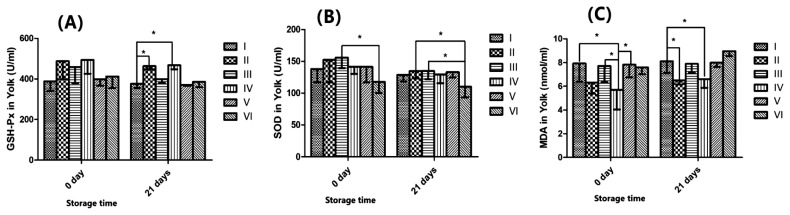
I-0 *Agaricus bisporus* stem residue (ABSR) supplement. II-2.0% ABSR supplement. III-4.0% ABSR supplement. IV-6.0% ABSR supplement. V-8.0% ABSR supplement. VI-10.0% ABSR supplement. (A) Glutathione peroxidase (GSH-Px) of yolk in two periods (0 d and 21 d) of treatments (mean and standard deviation [SD], n = 4, p<0.05 is marked as * on lines). (B) Superoxide dismutase (SOD) of yolk in two periods (0 d and 21 d) of treatments (mean and SD, n = 4, p<0.05 is marked as * on lines). (C) Malonaldehyde (MDA) of yolk in two periods (0 d and 21 d) of treatments (mean and SD, n = 4, p<0.05 is marked as * on lines).

Figure 2A indicates that ABSR had a significant effect on GSH-Px in yolk after 21 d storage. Treatment with 2.0% ABSR and 6.0% ABSR diet were significantly higher than that of the control treatment (p<0.05). We can see from Figure 2B that ABSR did not change SOD in yolk evidently (p>0.05). Figure 2C showed that ABSR can obviously abate the MDA in yolk (p<0.05) from 0 d until 21 d.

## DISCUSSION

Nowadays more and more research focuses on improving the health of laying hens through feeding natural functional ingredients or plant-based extracts. Results of this experiment show that ABSR has no significant effect on the production performance. This result is similar to some previous studies regarding laying hens fed with fungi [10–12]. Surprisingly, the application of 2% ABSR can significantly improve CP digestibility and total energy digestibility. This may be related to amelioration microbial population structure in the gut by ABSR. Another reason may be that the protein and carbohydrate in ABSR are more easily absorbed by birds, resulting in the improvement of CP and total energy digestibility. Lee et al [13] also showed that the addition of 3% to 5% fermented *Flammulina velutipes* mycelium could reduce the emission of ammonia in the feces of laying hens. The improvement of CP digestibility can reduce nitrogen emission, which is beneficial to environmental protection.

The results showed that there were no significant differences in egg quality and chamber height with time. The preservation effect of eggs is closely related to the quality of eggshell. The better quality of eggshell can effectively prevent the invasion of external bacteria [14–16]. This result may be due to ABSR having no significant effect on the permeability of eggshell. However, some indexes of the experimental treatment had a better trend than that of the control treatment. It can be supposed that with the extension of time, significant influence may appear.

*A. bisporus* contains a well-known carbohydrate, mushroom polysaccharide ((1→3)-α-glucans and (1→3), (1→6)-β-glucans, etc.). Mushroom polysaccharides showed high antioxidant activity in many *in vivo* and *in vitro* experiments [17,18]. Interestingly, previous research [19] showed that purified mushroom polysaccharides had no obvious antioxidant activity, and their antioxidant activity mainly depended on phenols and proteins, suggesting that polysaccharides, phenols and proteins may have synergistic reaction. Phenolic acids account for the highest percentage of phenolic compounds in mushrooms [20], including protocatechuic acid, gallic acid, p-hydroxybenzoic acid, etc. Phenolic acid can provide electrons to neutralize ROS and protect cells from damage. Phenolic acids can also chelate metals that can produce ROS, reducing the content of ROS in the body. Besides, ergothioneine, an antioxidant, is a key substrate of organic cationic transporters and plays a protective role in cells. In addition, the substance also has anti-mutagenesis and radiation protection activities. Some researchers have found that the content of ergothionein in fruiting bodies of *A. bisporus* was as high as 0.93 mg/g dry matter [21]. *A. bisporus* belongs to basidiomycetes. Besides, ergosterol, which exists in the fruiting bodies of basidiomycetes, is the most common antioxidant: fungal sterol. Some studies have found that ergosterol peroxide is a sterol compound responsible for antioxidant activity, and the trend of inhibiting lipid peroxidation increases with the increase of its concentration [22]. Vitamins such as ascorbic acid, carotenoids, thiamine, riboflavin, biotin and pyridoxine are also abundant in *A. bisporus* [23]. These vitamins are also favorable to the body’s antioxidant capacity. In addition, basidiomycetes mushrooms can effectively accumulate trace elements. *A. bisporus* is also rich in trace elements such as copper, zinc and selenium. The most common SOD is a copper-zinc complex enzyme, which participates in the elimination of free radicals in the body and catalyzes the degradation of superoxide anions into hydrogen peroxide or molecular oxygen. Supplementation of copper and zinc can increase the synthesis of SOD and enhance the antioxidant capacity of the body. Selenium also plays an important role in the body’s antioxidant systems. Some researchers have found that the antioxidant ability of laying hens increased significantly, and some antioxidants could be deposited in eggs when diets were supplemented with different forms of selenium [24]. All of the above-mentioned nutrients may be responsible for the improvement of antioxidant performance.

Giannenas et al [5] studied the effects of 1% and 2% fruiting bodies of *A. bisporus* on broilers. The results showed that the addition of these two levels increased the antioxidant capacity of tissues, significantly reduced the content of malondialdehyde, and increased the content of glutathione, glutathione reductase, GSH-Px and glutathione S-transferase. Giannenas et al [25] studied the effects of fruiting bodies of *A. bisporus* on turkeys. The results showed that the addition of 1% and 2% dry fruiting bodies of *A. bisporus* could significantly improve the antioxidant capacity of turkeys. Shang et al [26] found that adding *Hericium erinaceus* fermentation concentrate in broiler diet could significantly improve the antioxidant capacity of broilers. Lee et al [27] found that dietary supplementation of 1% and 2% dried *Pleurotus eryngii* stem residue could significantly improve serum antioxidant capacity. The above experimental results are similar to those of this experiment. This may indicate that the fruiting bodies of *A. bisporus* and ABSR may have similar effects. Moreover, the reason for these similar results may be related to secondary metabolites of fungi. Wang et al [28] found that secondary metabolites such as cordycepin, cordycepin acid, and phenolic compounds contained in *Cordyceps sinensis* could improve the oxidative status of laying hens and thus obtain better production performance.

Antioxidant capacity affects the health of laying hens, thus affecting the shelf life of laying hens. Antioxidant capacity is closely related to disease resistance. For example, fatty liver hemorrhage syndrome in laying hens is caused by lipid peroxidation of liver [29]. Improving the antioxidant capacity of laying hens can reduce the occurrence of fatty liver [30]. With the increase of antioxidant content in eggs, eggs should command a higher price than ordinary eggs. Eggs containing higher antioxidants can improve immunity of consumers as well as enrich nutrition. Furthermore, a waste product in *A. bisporus* production can now be used.

## CONCLUSION

In conclusion, supplementing ABSR can improve the utilization of protein and energy, increase the antioxidant substances in serum and yolk thus improve the antioxidant capacity of laying hens without affecting the laying performance and egg storage. ABSR can be a natural potential health promoter for laying hens. Based on the results of this study, we recommend the use of approximately 5% of ABSR in laying hens’ diets.

## Figures and Tables

**Table 1:** Nutrient levels of *Agaricus bisporus* stem residue (dry matter)

Item1)	Nutrient level (%)
Crude ash	8.12
Crude protein	28.26
Crude fat	1.33
Calcium	4.54
Phosphorus (total)	0.15

1) The nutrient content were measured values.

**Table 2:** Dietary composition and nutrient level of experiment dicts

Item	0 ABSR	2.0% ABSR	4.0% ABSR	6.0% ABSR	8.0% ABSR	10.0% ABSR
Ingredient (%)						
Corn	65.05	63.80	62.55	61.15	59.90	58.61
ABSR	0.00	2.00	4.00	6.00	8.00	10.00
Soybean meal	22.54	21.59	20.64	19.74	18.74	17.73
Soybean oil	0.70	0.90	1.10	1.40	1.60	1.85
Shell powder	4.55	4.55	4.55	4.55	4.55	4.55
Limestone	3.35	3.35	3.35	3.35	3.35	3.35
Paprika	0.30	0.30	0.30	0.30	0.30	0.30
Sodium selenite	0.01	0.01	0.01	0.01	0.01	0.01
Dicalcium phosphate	1.00	1.00	1.00	1.00	1.00	1.00
Premix1)	2.50	2.50	2.50	2.50	2.50	2.50
Lysine hydrochloride	0.00	0.00	0.00	0.00	0.05	0.10
Total	100	100	100	100	100	100
Nutrient levels2)						
AME (Kcal/kg)	2,718.68	2,718.68	2,718.68	2,718.68	2,718.68	2,718.68
CP (%)	16.81	16.81	16.80	16.80	16.81	16.81
Calcium (%)	3.62	3.62	3.61	3.61	3.60	3.59
Total phosphorus (%)	0.64	0.64	0.63	0.62	0.61	0.61
Digestible Lys (%)	0.84	0.81	0.78	0.76	0.77	0.78
Digestible Met (%)	0.39	0.38	0.38	0.37	0.36	0.36

ABSR, *Agaricus bisporus* stem residue; AME, avian metabolizable energy; CP, crude protein; Lys, lysine; Met, methionine.

1) Premix per kg: Vit A 340,000 IU; Vit D_3_ 150,000 IU; Vit E 700 IU; Vit K_3_ 135 mg; Vit B_1_ 80 mg; Vit B_2_ 180 mg; Vit B_6_, 130 mg; Vit B_12_ 700 μg; biotin 4,200 μg; nicotinamide 1,150 mg; folic acid 50 mg; calcium pantothenate 340 mg; choline 24 g; Cu 800 mg; Fe 9 g; Mn 4.5 g; Zn 4.5 g; I 120 mg; Se 14 mg; sodium chloride 12%.

2) Nutrient levels are calculated values.

**Table 3:** Effect of ABSR on performance of laying hens1)

Item	0 ABSR	2.0% ABSR	4.0% ABSR	6.0% ABSR	8.0% ABSR	10.0% ABSR	SEM	p-value		

								ANOVA	Linear	Quadratic
Hen-day egg production (%)	72.91	76.93	75.49	75.94	71.12	74.95	0.744	0.278	0.621	0.738
Average egg weight (g)	57.43	57.41	57.10	57.33	56.75	57.16	0.207	0.955	0.479	0.765
Daily feed intake (g/bird·d)	91.57^ab^	91.36^ab^	92.95^ab^	93.93^a^	91.03^b^	91.07^b^	0.374	0.120	0.754	0.129
FCR (feed/egg, g/g)	2.26	2.12	2.17	2.25	2.30	2.17	0.256	0.338	0.729	0.942

ABSR, *Agaricus bisporus* stem residue; SEM, pooled standard error of the means; ANOVA, one-way analyses of variance; FCR, feed conversion ratio.

1) Data represent the mean value of 4 replicates each treatment.

a,b Mean values within the same column with no common superscripts differ significantly (p<0.05).

**Table 4:** Effect of ABSR on nutrient digestibility of laying hens1)

Item	0 ABSR	2.0% ABSR	4.0% ABSR	6.0% ABSR	8.0% ABSR	10.0% ABSR	SEM	p-value		

								ANOVA	Linear	Quadratic
CP	33.98^b^	37.10^a^	35.99^ab^	36.96^ab^	35.60^ab^	36.41^ab^	0.399	0.228	0.304	0.223
Total energy	76.74^b^	78.50^a^	78.36^a^	78.60^a^	78.57^a^	77.16^ab^	0.245	0.060	0.624	0.008
Ca	46.35	47.58	45.77	44.97	46.00	46.74	0.430	0.678	0.699	0.631
Total P	23.95	24.37	24.16	22.93	22.60	23.84	0.455	0.887	0.462	0.727

ABSR, *Agaricus bisporus* stem residue; SEM, pooled standard error of the means; ANOVA, one-way analyses of variance; CP, crude protein.

1) Data represent the mean value of 4 replicates each treatment.

a,b Mean values within the same column with no common superscripts differ significantly (p<0.05).

**Table 5:** Estimation of suitable ABSR supplement level based on quadratic regression model1)

Item	Quadratic regression equation2)	R^2^	p-value	Optimal ABSR level3) (%)
Apparent digestibility of total energy (%)	Y = −0.069X^2^+0.726X+76.887	0.477	0.008	5.261
GSH-Px in serum (U/mL)	Y = −6.051X^2^+60.163X+352.432	0.320	0.038	4.971

ABSR, *Agaricus bisporus* stem residue; R^2^, coefficient of determination; GSH-Px, glutathione peroxidase.

1) According to the calculation of quadratic p-values, the item of significance (p<0.05) is selected to establish the quadratic regression equation.

2) In the equation, Y represents the value of the item, and X represents the ABSR supplement level.

3) In the quadratic regression equation (Y = AX^2^+BX+C), when Y reaches the maximum value, X = −B/2A.

**Table 6:** Effects of ABSR on antioxidant indexes in serum of laying hens1)

Item	0 ABSR	2.0% ABSR	4.0% ABSR	6.0% ABSR	8.0% ABSR	10.0% ABSR	SEM	p-value		

								ANOVA	Linear	Quadratic
GSH-Px (U/mL)	390.73^bc^	463.67^b^	541.01^a^	554.88^a^	364.99^c^	402.76^bc^	15.487	0.000	0.978	0.038
SOD (U/mL)	109.26^c^	149.25^a^	143.36^ab^	154.72^a^	153.52^a^	118.71^bc^	4.604	0.004	0.190	0.068
MDA (nmol/mL)	8.03	6.88	7.35	7.46	7.22	7.35	0.219	0.748	0.913	0.162

ABSR, *Agaricus bisporus* stem residue; SEM, pooled standard error of the means; ANOVA, one-way analyses of variance; GSH-Px, glutathione peroxidase; SOD, superoxide dismutase; MDA, malonaldehyde.

1) Data represent the mean value of 8 hens each treatment

a–c Mean values within the same column with no common superscripts differ significantly (p<0.05).
